# Impulsivity and Depressive Brooding in Internet Addiction: A Study With a Sample of Italian Adolescents During COVID-19 Lockdown

**DOI:** 10.3389/fpsyt.2022.941313

**Published:** 2022-07-11

**Authors:** Pierluigi Diotaiuti, Laura Girelli, Stefania Mancone, Stefano Corrado, Giuseppe Valente, Elisa Cavicchiolo

**Affiliations:** ^1^Department of Human Sciences, Society and Health, University of Cassino and Southern Lazio, Cassino, Italy; ^2^Department of Human, Philosophical and Educational Sciences, University of Salerno, Fisciano, Italy

**Keywords:** Internet addiction, adolescents, COVID-19, motor impulsivity, attentional impulsivity, ruminative thinking, depression, gender

## Abstract

This contribution presents a study conducted on a sample of Italian adolescents (*n* = 411) in the period of the first COVID-19 lockdown. The study investigated the role and predictive weight of the impulsivity and depressive brooding variables on Internet addiction, using a hierarchical regression analysis. The participants were administered the *Uso-Abuso e Dipendenza da Internet* [Internet Use-Abuse and Addiction] (UADI-2), the *Barratt Impulsiveness Scale-11* (BIS-11), and the *Ruminative Response Scale* (RRS). In terms of percentage distribution, 28% of the participants were in the full dependency range, while 34.7% demonstrated Internet abuse behavior. The results highlighted not only the predictive value of impulsiveness (β = 0.323) and ruminative thinking (β = 0.258), but also the role of gender (β = −0.205) on Internet addiction. Thus, male participants showed higher levels of Internet addiction, with higher scores on impulsiveness and brooding way of thinking. The study shows that the issue in question is significantly present among adolescents; in addition, not only targeted awareness programmes but also psycho-educational and clinical interventions to promote greater emotional and cognitive control would be necessary as a preventive and mitigating measure. Psychological interventions can help increase self-awareness, develop emotional regulation and impulse control, and correct maladaptive cognitions which in adolescents are mostly driven by a ruminative cognitive style.

## Introduction

Internet addiction in adolescence can be a real syndrome: it affects boys and girls who cannot do without it and, deprived of the web, feel a strong discomfort that they cannot alleviate in any other way. Mobile phones and digital technologies, especially during adolescence, have increasingly taken on a social role. They are an integral part of adolescents' lives as tools for communication and relationships, information, study, creativity and, above all, for participation (1–3). A recent study showed that 5% of 14–21 year-olds are moderately dependent on the Internet and 0.8% are seriously dependent (4). It is pointed out that online addiction can include gaming, shopping, social networks but especially porn sites. Internet addiction constitutes a psychological symptom that can be connected to different diagnostic and clinical frameworks (5–8).

Addiction occurs when most of the time and energy are spent in using the network, thus creating strong and dysfunctional impairments in the main and fundamental existential areas, such as personal, relational, educational, family and emotional (9–13). Recent studies have reported adolescents who spend sleepless nights repeatedly checking their mobile phone, which is compulsorily switched on 24 h a day (14–18).

Internet Addiction Disorder (IAD) has received increasing attention from the scientific community (19–24). Moreover, there has also been a growing concern among the public and the national health authority regarding the health and societal costs of the Problematic Use of the Internet (PUI) across the lifespan. As effectively highlighted by Fineberg et al. (25), further research is needed to advance the understanding of PUI, with the aim of identifying vulnerable individuals for early intervention and supporting a strengthened European network in order to create collaborative research networks, shared multinational databases, and multicenter studies.

Recently, there has been an increasing use of the term smartphone addiction risk (26–28). According to Greenfield and Davis (29), attachment to smartphones is very similar to all other addictions in that it causes interference in the production of dopamine, the neurotransmitter that regulates the brain's reward circuitry: in other words, it encourages people to perform activities that they believe will give them pleasure. So every time they see a notification appear on their mobile phone, their dopamine levels rise because they think something new and interesting is in store. But the problem is that there is an urge to check it over and over again, triggering the same mechanism that is activated in a gambler (30).

Numerous studies link Internet addiction to various health problems affecting individuals and their way of relating to the world around them. Particularly alarming, however, is the relationship between Internet addiction and depression or depressive symptoms (31, 32).

Internet addiction can be particularly dangerous for people exposed to negative clinical outcomes, such as suicidality. Individuals with suicidal thoughts could be more likely to use the Internet to search for suicide-related information (33). For example, using samples of European teenagers with an average age of 15 years, Kaess et al. (34) and Sami et al. (35) discovered that pathological Internet usage is associated to a variety of mental issues, including suicide thoughts and depression. In a Spanish cohort of teenagers aged 13–17, Gámez-Guadix et al. (36) investigated the relationship between depressive illness and elements of problematic Internet usage. According to Banjanin et al. (37), the amount of Internet addiction is linked to depressed symptoms among Serbian adolescents aged 13–17. Several studies, however, have found a mixed association between addiction and depression. In this regard, various scholars have demonstrated that addiction has an impact on depressed symptoms (38–40). The inverse is also true: depressed symptoms anticipate addiction, thus people with depression use their phones to cope with their unpleasant emotions (41, 42). Furthermore, lonely teens may find it more difficult to form face-to-face connections, which may boost their interest in online dating (41). Therefore, there is no consensus on which variable is dependent and which is independent. Several studies agree that excessive Internet use is linked to impulsivity and depression, as the individual, when trying to regulate his or her emotions, is unable to control them and chooses to regulate them in a negative way, i.e., through an activity that harms him or her, such as problematic Internet use (43, 44).

Rumination, according to Davis (45), is an underpinning aspect in Internet addiction, since it generates persistent thinking about concerns relating to Internet use rather than other events in one's life. These ruminative impulses may lead to the reinforcing of Internet-related associations. As a result, those who have a lot of ruminating may have a more severe and long-lasting Internet addiction. Other recent studies have investigated both the relationship between Internet addiction and general rumination (46–50) as well as the specific one with so-called social media rumination, that is the propensity to continually ponder about one's social media postings, relevant situational conditions, and the ramifications of those posts, displaying a recursive cycle process with mental anguish (51–55).

## The Present Study

With reference to the above literature, there is still a lack of studies that investigate and confirm the role of individual antecedents on problematic and pathological Internet use in socially isolated adolescents. Therefore, the present study aimed to assess the predictive role of impulsivity and depressive rumination in a sample of Italian adolescents during the COVID-19 pandemic lockdown period of 2020.

We expected that high levels of Internet addiction would be associated with similarly high levels of impulsivity and depressive brooding.

## Materials and Methods

### Participants

The survey included 411 high school students, with 201 (48.9%) boys and 210 (51.1%) girls between the ages of 16 and 18, with an average age of 17.85 and SD of 1.12. The data collection was carried out following parental consent and approval of the research project by the school board. The administration took place on October and November 2020, collectively, by compiling a digital protocol in the computer rooms of the institutes involved in the research, in the presence of the psychologists and the teachers responsible for the class. Anonymity and the use of aggregate data for research purposes were guaranteed to participants. They also signed a permission form giving their written informed agreement to take part in the study. The procedure was authorized by the local University Institutional Review Board.

### Tools

- *Uso-Abuso e Dipendenza da Internet* [Internet Use-Abuse and Addiction] [UADI-2; (56)], assesses the psychopathological risk of Internet abuse and the psychological use that users make of the network by means of 24 items. Replies for each item were chosen from a 5-point scale ranging from 1 (absolutely false to me) to 5 (absolutely true for me). The UADI-2 assesses four dimensions: Dissociation, Impact on Real Life, Addiction Symptoms, Identity and Sexuality. The scoring has three score ranges: up to 62, normal internet use; 63–74, Internet abuse; over 74, Internet addiction. Cronbach's alpha was 0.823.
- *Barratt Impulsiveness Scale-11* [BIS-11; (57); Italian version: (58)] is a 30-item self-report questionnaire that measures general impulsivity while accounting for the multidimensional character of the construct. The scale includes six first-order factors (attention, motor, self-control, cognitive complexity, perseverance, and cognitive instability) and three second-order factors: attentional impulsivity, motor impulsivity (motor and perseverance), unplanned impulsivity (self-control and cognitive complexity). The total score is obtained by summing the first and second-order factors. Items range from 1 (rarely/never) to 4 (always). Cronbach's alpha was 0.786.
- *Ruminative Response Scale* [RRS, (59), Italian validation (60)]. The RRS measures individuals' general tendency to ruminate (61–63) by means of 22 items. The items tap different aspects of rumination and they are rated on a 4-point scale from 1 (never) to 4 (always). Cronbach's alpha was 0.911.

### Statistical Analysis

Descriptive analysis (percentages, means, standard deviation, skewness and kurtosis, confidence intervals); *t*-test for comparison of scores with respect to gender; Pearson's bivariate correlations; testing of univariate and multivariate regression assumptions; hierarchical regression; Cohen's *d* as effect size measure (0.20 = small, 0.50 = medium, and 0.80 = large).

## Results

Descriptively, 28.0% (*n* = 113) of the participants were highly dependent on internet (with a mean score on the UADI-2 > 74). The 34.7% (*n* = 143) of respondents were found to be in the net abuse range (with a mean score between 63 and 74). The remaining 37.3% (*n* = 155) were in the normal range of network use. Among males, 35.8% (*n* = 72) are addicted to the network, while 36.8% (*n* = 74) have network abuse behavior. Among females, 19.5% (*n* = 41) are addicted while 30.9% (*n* = 65) abuse the network. The gender comparisons are shown in Table 1 where the *t*-tests between the two groups and the respective breakdowns in the range of full dependency, abuse and normal Internet use are reported. It is clear to observe that no significant gender differences emerged in the respective bands.

**Table 1:** Differences in the level of Internet addiction with respect to gender of participants.

**Measures**	**Males**	**Females**			
Dependence	M (SD)	M (SD)	CI 95%	*p*	d
	83.44 (6.35)	82.56 (5.47)	[−1.46; 3.23]	*>0.05*	0.15
Abuse	M (SD)	M (SD)	CI 95%	*p*	d
	67.56 (3.22)	67.89 (3.09)	[−1.37;0.722]	*>0.05*	0.10
Normal use	M (SD)	M (SD)	CI 95%	*p*	d
	53.93 (6.26)	53.56 (5.49)	[−1.67; 2.41]	>0.05	0.06

*p is significant if < 0.05; d = Cohen's d*.

In Table 2 it can be seen that the level of male dependence is higher both in terms of the overall score and in relation to the subscales of Dissociation, Identity and Sexuality and Impact on Real Life, whereas the manifestation of addiction symptoms did not differ significantly between genders.

**Table 2:** General and detailed Internet addiction with respect to gender of participants.

**Measures**	**Males**	**Females**			
General addiction	M (SD)	M (SD)	CI 95%	*p*	d
	70.03 (12.42)	63.86 (12.11)	[3.79; 8.55]	* <0.001*	0.50
Dissociation	M (SD)	M (SD)	CI 95%	*p*	d
	20.55 (3.97)	18.62 (4.25)	[1.13; 2.73]	* <0.001*	0.46
Real Life Impact	M (SD)	M (SD)	CI 95%	*p*	d
	13.94 (2.62)	13.57 (2.60)	[−0.132;0.879]	>0.05	0.14
Addiction Symptoms	M (SD)	M (SD)	CI 95%	*p*	d
	22.41 (5.46)	21.60 (5.26)	[−0.237; 1.84]	>0.05	0.15
Identity and Sexuality	M (SD)	M (SD)	CI 95%	*p*	d
	13.13 (4.67)	10.07 (4.57)	[2.17; 3.96]	<0.001	0.66

*p is significant if < 0.05; d = Cohen's d*.

The descriptive values of all the variables utilized in the study are presented in Table 3.

**Table 3:** Descriptive values of the variables.

	**Skewness**	**(*SE*)**	**Kurtosis**	**(*SE*)**	**Mean**	**(*SD*)**
Age	−0.272	0.120	−0.518	0.240	17.85	1.12
General internet addiction	0.206	0.120	−0.400	0.240	66.88	12.63
Dissociation	−0.058	0.120	−0.350	0.240	19.57	4.22
Real life impact	0.233	0.120	−0.064	0.240	13.75	2.61
Addiction symptoms	−0.012	0.120	−0.392	0.240	21.99	5.37
Identity and sexuality	0.306	0.120	−0.844	0.240	11.57	4.86
Ruminative response	0.109	0.120	0.294	0.240	51.86	11.19
Total impulsivity	−0.625	0.120	0.235	0.240	69.31	9.81
Motor impulsivity	−0.100	0.120	−0.345	0.240	23.42	5.10
Attentional impulsivity	−0.108	0.120	−0.234	0.240	18.17	3.31
Non-planning	−0.387	0.120	1.18	0.240	27.71	4.10

*SE, Standard Error; SD, Standard Deviation*.

The bivariate correlations between the study's measurements are shown in Table 4. It can be observed that there are significant associations with both the Ruminative Response subscale (0.375 with *p* < 0.01) and the Impulsivity subscale (0.437 with *p* < 0.01). More specifically for the latter measure, Internet addiction reported correlations with the subscale of attentional impulsiveness (0.392 with *p* < 0.01) and motor impulsiveness (0.481 with *p* < 0.01), while the correlation with the subscale of non-planning (0.131 with *p* < 0.01) was lower.

**Table 4:** Bivariate correlations.

**General internet addiction (UADI-2)**	**1**	**2**	**3**	**4**	**5**	**6**	**7**	**8**	**9**	**10**
Dissociation (UADI-2)	0.821**	1						.		
Real life impact (UADI-2)	0.301**	0.183**	1							
Addiction symptoms (UADI-2)	0.779**	0.486**	−0.085	1						
Identity and sexuality (UADI-2)	0.864**	0.629**	0.181**	0.544**	1					
Ruminative response (RRS)	0.375**	0.273**	0.012	0.387**	0.303**	1				
Total impulsivity (BIS-11)	0.437**	0.391**	0.206**	0.217**	0.445**	0.351**	1			
Motor impulsivity (BIS-11)	0.481**	0.413**	0.205**	0.282**	0.468**	0.411**	0.859**	1		
Attentional impulsivity (BIS-11)	0.392**	0.316**	0.117*	0.243**	0.411**	0.410**	0.791**	0.624**	1	
Non-planning (BIS-11)	0.131**	0.166**	0.142**	−0.028	0.149**	−0.002	0.683**	0.308**	0.310**	1
Age	−0.048	−0.072	0.017	0.010	−0.047	0.006	−0.063	−0.060	−0.071	−0.039

N = 411; **Correlation is significant at the 0.01 level (2-tailed). *Correlation is significant at the 0.05 level (2-tailed). For Age Spearman's correlation has been used. Pearson's for the other variables.

In order to identify predictors of Internet addiction, a hierarchical regression was performed on the variables of ruminative response and impulsivity. The presence of multivariate outliers was ruled out in the early verifications of the regression assumptions. Mardia's multivariate kurtosis index (104.59) was below the crucial value [p (p + 2) = 143], implying that the connection between the variables is largely linear. Little variance inflation factor (VIF) values 2 and high tolerance values > 0.60 showed low co-linearity. The average of the standardized and raw residuals was set to 0 for verification of the residual assumptions; the Durbin–Watson test resulted in a value of 1.98, indicating the lack of autocorrelation.

The inclusion of Ruminative Response, Impulsivity, and Gender to a hierarchical multiple regression was used to see if it enhanced the prediction of Internet addiction. The full model resulted statistically significant, *R*^2^ = 0.288, *F*_(3, 410)_ = 54.948, *p* < 0.001; adjusted *R*^2^ = 0.283.The regression model included Impulsivity and Ruminative Response at step 1, Gender at step 2. Table 5 shows the results of the hierarchical multiple linear regressions. In the regression model, with Internet addiction as outcome variable, Impulsivity and Ruminative Response jointly explained a 25% portion of the outcome variability. Including Gender in the second step resulted in a considerable increase in the explained variance, which improved to 29%. Standardized beta values were significant with a positive sign for Impulsivity and Ruminative Response, and a negative sign for Gender.

**Table 5:** Results of hierarchical linear regression analyses.

**Outcome variable**			
**Internet addiction**			
**Independent variable**	**Adjust**. ***R***^**2**^	***ΔR*** ^**2**^	***β***
Step 1	0.243***	0.247***	
Impulsivity			0.348***
Ruminative response			0.253***
Step 2	0.283***	0.041***	
Impulsivity			0.323***
Ruminative response			0.258***
Gender			−0.205***

N = 411; β = standardized beta value. ***p ≤ 0.001.

In order to evaluate the weight in the model of the different factors that make up impulsivity, further hierarchical regressions were carried out by alternately including the variables of attentional impulsivity, motor impulsivity and non-planning. The regression with the last variable (non-planning) did show a significant weight and explanatory value of Internet addiction. Therefore, the two models with the alternative inclusion of motor and attentional impulsivity are reported below. In the first case, the full model resulted statistically significant, *R*^2^ = 0.247, *F*_(3, 410)_ = 44.485, *p* < 0.001; adjusted *R*^2^ = 0.241.The regression model included Attentional Impulsivity and Ruminative Response at step 1, Gender at step 2. Table 6 shows the results of the hierarchical multiple linear regressions. In the regression model, with Internet addiction as outcome variable, Attentional Impulsivity and Ruminative Response jointly explained a 21% portion of the outcome variability. Adding Age at the second step provided a significant improvement in the explained variance, which reached 25%. Standardized beta values were significant with a positive sign for Attentional Impulsivity, Ruminative Response, and a negative sign for Gender.

**Table 6:** Results of hierarchical linear regression analyses inserting attentional impulsivity.

**Outcome variable**			
**Internet addiction**			
**Independent variable**	**Adjust**. ***R***^**2**^	***ΔR*** ^**2**^	***β***
Step 1	0.205***	0.208***	
Attentional impulsivity			0.286***
Ruminative response			0.256***
Step 2	0.241***	0.038***	
Attentional impulsivity			0.247***
Ruminative response			0.271***
Gender			−0.199***

N = 411; β = standardized beta value. ^***^p ≤ 0.001.

In the second case, the full model resulted statistically significant, *R*^2^ = 0.307, *F*_(3, 410)_ = 60.072, *p* < 0.001; adjusted *R*^2^ = 0.302. The regression model included Motor Impulsivity and Ruminative Response at step 1, Gender at step 2. The results of the hierarchical multiple linear regressions are presented in Table 7. In the regression model, with Internet addiction as outcome variable, Motor Impulsivity and Ruminative Response jointly explained a 27% portion of the outcome variability. When Gender was added in the second step, the explained variance went up to 31%. Standardized beta values were significant, with a positive sign for Motor Impulsivity, Ruminative Response, and a negative sign for Gender.

**Table 7:** Results of hierarchical linear regression analyses inserting motor impulsivity.

**Outcome variable**			
**Internet addiction**			
**Independent variable**	**Adjust**. ***R***^**2**^	***ΔR*** ^**2**^	***β***
Step 1	0.265***	0.269***	
Motor impulsivity			0.393***
Ruminative response			0.214***
Step 2	0.302***	0.038***	
Motor impulsivity			0.365***
Ruminative response			0.222***
Gender			−0.196***

N = 411; β = standardized beta value. ***p ≤ 0.001.

## Discussion

The results of the study report a significant proportion of adolescents in the addiction phase (one third of the total sample), while another third of the sample demonstrates network abuse behavior. Considering that the administration took place during the period of greatest impact of the COVID-19 pandemic, which as we know imposed prolonged isolation and rarefied direct contacts, it is likely that these high percentages suffer from the impact of social isolation on mental health and wellbeing [e.g., (64–68)] and that this contributed to a compensatory search in the network. However, the results are similar to those found in studies such as Putri et al. (69), Serra et al. (70), Paschke et al. (71), Servidio et al. (72), and Bhatia (73), which were also conducted with adolescents during the lockdown period. Useful data on the Italian COVID-19 lockdown and its impact on addictive behavior in general can be found in Martinotti et al. (74).

Most studies show a generally massive increase in media consumption by adolescents during lockdown, with or without pre-existing mental illness. In the forced form of preemptive “isolation,” a vicious circle is created that pushes people to seek comfort, entertainment, distraction and relief online, putting aside contingent discomforts (75–77). Previous and contemporary research has shown that teenagers' time perspectives may be engaged in the state of internet addiction, which reinforces procrastination practices that severely reduce their academic performance (78–81).

Our results highlight the male prevalence of Internet addiction, in line with other studies carried out with adolescents during the same period (72, 82, 83). Regarding gender differences, the literature indicates that males are generally attracted to sex sites and online games. Females are more likely to spend time flirting in chat rooms. Men prefer visual cues and explicit sexual encounters, while women are more focused on bonding and interactions (84–87). These aspects are congruent with the findings of the gender comparisons of the component factors of the UADI-2 dependency scale. The significantly higher score on the dissociation scale for males is associated with increased gaming [see also (7, 8, 88, 89)], whereas the score on the identity and sexuality scale is more likely to relate to behavior related to searching the web for sexually oriented content or masking one's identity in chat rooms or role-playing games [see also (90–92)]. While no gender differences were found with regard to the manifestation of specific addiction-related symptoms, the negative impact on real life (study, social relationships, and general wellbeing) was nevertheless greater for males.

The analysis of bivariate correlations clearly confirmed both the association with impulsivity and that with depressive brooding. It is noteworthy that for the subscales of impulsivity, the tendency not to plan is not associated with Internet addiction. Considering the other two subscales, motor impulsivity and attentional impulsivity, in isolation, the magnitude of the association is even greater than the overall measure of impulsivity.

The subsequent hierarchical regression also confirmed the hypotheses of the present study. In terms of the weight of the regression coefficients, impulsivity remains the main predictor (β = 0.323), as indicated by most of the literature cited above, but it is flanked by rumination, which shows a regression weight just below the former (β = 0.258).

As reported in Bagatarhan and Siyez (49), rumination is defined as the repetition of thoughts that arise involuntarily, prevent the individual from acting, are primarily transient, difficult to control, and require significant effort to stop (93). Rumination has been defined both as a maladaptive method of emotion management (e.g., an attempt to gain an understanding of one's dysphoric state of mind while lacking active solutions to the problem) (94) and as a metacognitive process (95).

It is crucial to examine rumination in adolescence since it is at this period of life that teenage egocentrism arises, granting adolescents the ability for meta-cognition, including self-consciousness and self-reflection; these capacities enhance the likelihood of rumination (96). Mental rumination can be considered an emotional regulation strategy that is often used in anger experiences (97). Rumination and brooding involve many areas of thought and provoke different behaviors. The three primary features are the dominance of verbal thought over visual imagination, cognitive avoidance, i.e., the failure to seek analysis, in-depth study, curiosity about the event; emotional inhibition in which the subject is deprived of and in fact becomes a prisoner of his own thoughts. Typical of brooding is the presence of a strong sense of vagueness and lack of concreteness. Predisposing factors for brooding are excessive vigilance, selective attention (seeking confirmation of one's ideas) and the presence of negative information from past experiences in long-term memory. Adolescent crises normally induce quite frequent phases of elaborative brooding. Given the role that rumination is thought to play in the onset of teenage depression (98–100), it seems important to study the specificity of this cognitive vulnerability factor in this particular age group. Some scholars have pointed out that the presence of rumination is often related to increased anger, embarrassment, and negative affectivity (101–103).

Both impulsivity and rumination are seen as transdiagnostic susceptibility factors in adolescents, contributing to the development and persistence of internalizing symptoms throughout adolescence (104, 105) and particularly among girls (106). Davis' (45) cognitive-behavioral model of pathological Internet use, which considers cognitive processes to explain the emergence of Internet addiction, highlights the association between rumination and Internet addiction. According to this paradigm, the most essential component of Internet addiction is maladaptive cognitions driven by a ruminative cognitive style. Rumination is the habit of obsessing over difficulties related to Internet use rather than other events in one's life. It appears that adolescents engage in the use of technology to prevent depressive, which can lead to overuse and similar to addiction symptoms (107).

Rumination, common in depressive symptoms (108), has also been studied in relation to harmful online behaviors mediated by technology. The recent contribution by Wang et al. (109) demonstrated that in teenagers, rumination mediated the association between social networking website addiction (SNS; i.e., problematic and compulsive online social networking) and depression, and that the mediating function was moderated by self-esteem. The immoderate and improper use of mobile phones as well as of the Internet can also lead adolescents to withdraw from others, to develop relational insecurities or to nurture a fear of rejection, to feel inadequate and in need of support, even if it is external and an end in itself. It should not be forgotten that among these forms of addiction, there is also what is known as ludopathy, i.e., addiction to games and gambling, to which mobile devices also contribute on a large scale (110–112).

According to Kircaburun et al. (107), mindfulness and rumination have a mediation role in the association between trait emotional intelligence and problematic social media use (PSMU) and problematic online gaming (POG). Teenagers with greater trait emotional intelligence and mindfulness exhibited less ruminating and, as a result, lower PSMU. In addition to reaffirming the correlation between rumination and Internet addiction, Bagatarhan and Siyez (49) demonstrate in a sample of teenagers that depression mediated the relationship between rumination and Internet addiction, but this resulted only for female adolescents. This conclusion, according to the authors, is consistent with previous research suggesting that depression in female adolescents is more effective than depression in male adolescents in the onset of Internet addiction [e.g., (113)]. However, other works have already explored the relationship between internet addiction, impulsivity and ruminant thoughts, also in Italian samples. Specifically, Di Carlo et al. (114) confirmed the role of impulsivity in the Problematic Use of Internet (PUI), while also emphasizing the association of obsessional impulses with this pathological behavior. They hypothesized a trigger role of obsessive impulses for the engagement in PUI, together with factors such as negative affective states.

Recently, Zhang et al. (115) analyzed the link between cell phone addiction and cognitive disorders in Chinese adolescents. They found a sequential mediation of the rumination → mindfulness effect that was also an important path through which mobile phone addiction affected cognitive failures in adolescents. Ruminant individuals over-process negative information, inducing negative moods and experiences in them; thereby reducing their ability to perceive present moment events and experiences (116). Hence, the sequential mediation of rumination → mindfulness represents a critical path through which mobile phone addiction affects cognitive failures in adolescents. The authors conclude by arguing that mobile phone addiction in adolescents has a direct impact on cognitive failures. To avoid such failures, adolescents need to train themselves to exercise more self-control in managing the use of mobile phones and curbing their desire to use the device excessively. Second, mobile phone addiction may disrupt the emotional balance and induce rumination, which leads to cognitive failures in task processing.

In line with the majority of the most recent international studies mentioned above, the results of our study also confirmed the predictive role of the impulsivity variables (motor and attentional) and that of depressive brooding with the addition of the Gender variable on a sample of Italian adolescents. Because Internet addiction is a frequent problem among teenagers, it is critical to create and execute Internet addiction prevention programs that are targeted to the unique requirements of adolescents, especially in light of the severe limits encountered during the pandemic (117).

The loneliness of remaining at home during the COVID-19 epidemic may have prompted teens to utilize mobile phones for amusement in order to alleviate the psychological feeling of isolation (118, 119). Both social loneliness (lack of intimate connections with others) (120) and emotional loneliness (which originates from not frequenting social circles) can generate unpleasant feelings toward oneself, such as despair and melancholy, which may cause people to overuse cell phones as a method of building relationships, satisfying the desire to belong, and mitigating negative feelings that arise from difficult circumstances (121).

Several studies have shown that during the pandemic adolescents' sleep quality was severely affected by the mobile phone use pattern, stress level, and sleep pattern, and that this can also weaken immune responses to infection (122–125). Teenagers with weak self-regulation and self-control would be unable to resist their inner need to use mobile phones (e.g., escape motive), which may lead to an uncontrolled rise in mobile phone usage time and, ultimately, to problematic mobile phone use (PMPU). Studies reveal that self-control moderates adolescent behavioral issues significantly, and teenagers with poor self-control are more likely to engage in problematic Internet usage (119, 126–130) and other negative outcomes (131, 132).

Psychological interventions can help reduce the intensity of Internet addiction by focusing on three main goals: reducing hours of use, improving functioning in critical areas of life, and minimizing exposure to harmful online information and activities (133). The most often utilized psychological treatment for Internet addiction is cognitive behavioral therapy (CBT), which is less intrusive than therapies for other addictive disorders. CBT methods for the treatment of Internet addiction in teenagers have been shown to be successful in lowering the symptoms of video game addiction in particular and Internet usage in general (134, 135). The following are the primary evidence-based CBT techniques for treating Internet addiction in adolescents: identifying the advantages and disadvantages of using the Internet; enhancing self-awareness, environmental awareness, and awareness of others; identifying and comprehending the precursors that lead to compulsive Internet consumption (e.g., disconnection from certain applications, complex emotional state, environmental changes, and key events); developing emotional regulation and behavioral inhibition in relation to Internet access (e.g., calming methods and relaxing muscles and breathing exercises); learning and implementing time management tactics; improving interpersonal and social communication skills; and devoting time to hobbies such as art, sports, and dancing (136–138).

Parenting practices are one of the aspects related to adolescents' Internet addiction. Some research has shown that parenting styles have an impact on problematic Internet use or Internet addiction in adolescents (139, 140). This approach would require training for parents to increase communication and problem-solving skills with their children (141). Because supportive family functioning was linked to a decreased risk of developing Internet addiction, family factors may also represent important targets for Internet addiction interventions (10).

Family therapy is not a single procedure, but rather a set of interventions aimed at strengthening family processes and bonds rather than directly addressing addictive behaviors. Treatments are designed to improve communication and relationships, transfer the fulfillment of psychological needs from Internet to interpersonal needs, and foster family ties (142, 143). Motivational interviewing and family-based treatment were merged by Shek et al. (144), and the results of their study showed that participants had a reduction in Internet addiction and better family functioning. However, it should be noted that most of the above-mentioned interventions are carried out with small groups of adolescents, so the extent of their effectiveness remains open to question. Further research and implementation of targeted and customized programmes will certainly be necessary.

### Strengths of the Study

The survey, carried out in south-central Italy during the COVID-19 pandemic lockdown, coincided with a particularly critical time in the relational lives of adolescents subjected to prolonged social isolation. It was therefore important to monitor the risk, especially in the most vulnerable individuals, of developing an addiction resulting from an excessive and spasmodic use of the Net as compensation for limitations in movement and direct contact with peers and in response to heavy frustrations and perceived discomforts in the emergency situation experienced.

Our research project had time to return the results to the teachers of the schools that collaborated in the study. An information and awareness-raising seminar for teachers was conducted in each of the participating institutions. Subsequently they were given the aggregate outline of the critical issues that emerged, with an illustration of the value of the predictors identified by the study (impulsivity and depression-type brooding) so that they would increase their attention to their joint remarks and interaction in their students. Teachers, in turn, were asked to raise not only the students' awareness of the theme of Internet addiction, but also their parents' awareness at regular school-family meetings, informing them of the critical issues that emerged through the project carried out and providing them with the contact details of the local health service unit dedicated to addiction issues (SERD), where specialized professionals (clinical psychologists and psychiatrists) would promptly take up any requests for support. Therefore, one merit of the study is that it not only monitored the progress of the internet addiction issue in the area, but also raised the awareness of teachers, pupils and parents with their active participation.

### Limitations of the Study

Our article has some limitations. First, the sample size for this study was small and the statistical power can be affected. This limitation was due to the difficulty in recruiting a large number of students during the COVID-19 pandemic lockdown period of 2020, when all courses were being taught remotely. To extend the applicability of our results, future studies could benefit from a larger sample size and the selection of participants from other parts of the country. Second, it might be interesting to include more variables, both related to individual background characteristics (such as socio-economic status) as well as clinical data (such as depression, anxiety, feeling of loneliness, interpersonal issues, and maladaptive cognitions), to investigate their relationships with Internet addiction. Moreover, the present study was based on cross-sectional data and future research is therefore needed in order to clarify the possible reciprocal influences between Internet addiction, impulsivity and depressive brooding. A further limitation was the use of an instrument for the internet addiction measure constructed and validated for the Italian adolescent population; in an international comparative data perspective, perhaps it would have been appropriate to include an internationally recognized instrument such as the Young's (19) *Internet Addiction Test* (IAT). A subsequent replication of the study with the same sample, after coming out of the most critical moment of the Coronavirus pandemic and coinciding with the return to in-person classroom activities for the learners, would have been appropriate in order to compare internet addiction trends longitudinally as well. Considering that the research project included the moment when the results were issued, a further limitation was the lack of a subsequent follow-up to monitor the response of the learners and their parents, and also to collect information from the local addiction service providers regarding what the request was for adolescent consultation and therapeutic intervention solicited by our initiative.

## Conclusion

Despite the above-mentioned limitations, we believe that this study makes an effective contribution to the literature. Firstly, our results shed light on the role and importance of impulsivity and depressive brooding on Internet addiction in a sample of Italian adolescents during the COVID-19 lockdown period, by means of a hierarchical regression analysis. Moreover, the present study takes into consideration differences between males and females in their use of Internet. This is an important aspect in order to plan interventions that focus on the specific needs of young people. Finally, we used well-established and reliable measures to test our hypotheses.

In the light of our findings, psycho-educational and clinical interventions are needed in order to encourage greater emotional and cognitive control in adolescents. The loneliness of remaining at home during the COVID-19 epidemic may have prompted teens to avoid reality using mobile phones, social media, and Internet contents for entertainment, but most of all to reduce the psychological experience of loneliness, anxiety, and stress. Adolescents who lack self-regulation and self-control may be unable to regulate their impulses, which may lead to an uncontrolled and problematic Internet use. Unfortunately, this behavior can represent a risk for adolescents even after the end of the COVID-19 pandemic and psychological therapies may help reduce the degree of Internet addiction. For example, in the context of Internet access, treatments could focus on increasing self-awareness, improving emotional regulation, impulse control, and correcting maladaptive cognitions, which in adolescents are mostly driven by a ruminative cognitive style.

## Data Availability Statement

The raw data supporting the conclusions of this article will be made available by the authors, without undue reservation.

## Ethics Statement

The studies involving human participants were reviewed and approved by Institutional Review Board (IRB) of the University of Cassino and Southern Lazio. The patients/participants provided their written informed consent to participate in this study.

## Author Contributions

PD, SM, and SC designed the study. PD, SM, SC, and GV analyzed the data and discussed the results. PD, EC, and LG drafted the manuscript. SM, GV, and SC revised the manuscript. All authors contributed to the article and approved the submitted version.

## Conflict of Interest

The authors declare that the research was conducted in the absence of any commercial or financial relationships that could be construed as a potential conflict of interest.

## Publisher's Note

All claims expressed in this article are solely those of the authors and do not necessarily represent those of their affiliated organizations, or those of the publisher, the editors and the reviewers. Any product that may be evaluated in this article, or claim that may be made by its manufacturer, is not guaranteed or endorsed by the publisher.
